# The muscle stem cell case of Benjamin Button: rejuvenating muscle regenerative capacity through nutraceuticals

**DOI:** 10.1172/JCI185054

**Published:** 2024-12-16

**Authors:** Taylor Peach, Mattia Quattrocelli

**Affiliations:** Molecular Cardiovascular Biology, Heart Institute, Cincinnati Children’s Hospital Medical Center and Department of Pediatrics, University of Cincinnati College of Medicine, Cincinnati, Ohio, USA.

## Abstract

Aging negatively affects the capacity of muscle stem cells (MuSCs) to regenerate muscle. In this issue of the *JCI*, Ancel, Michaud, and colleagues used a high-content imaging screen to identify nicotinamide and pyridoxine as promoters of MuSC function. The combination of the two compounds promoted MuSC function in vivo in aged mice and in primary cells isolated from older individuals. Furthermore, the two compounds were lower in the circulation of older men, paralleling decreases in lean mass and gait speed. These results advance the translational perspective of rejuvenating MuSC function through nutraceuticals.

## Background and main findings

Aging inevitably impacts our lives. One of the most distinctive signs of aging is sarcopenia, i.e., the loss of muscle mass and function that associates with older age, mobility loss, and reduced quality of life (1). Muscle stem cells (MuSCs) are no stranger to the aging-related detriments. Indeed, aging MuSCs fail to supply the skeletal muscle with the proper regenerative capacity, contributing to sarcopenia (2). Pharmacological strategies to rescue the aging MuSCs defects are therefore a compelling, yet unelucidated, avenue to investigate with the goal of counteracting sarcopenia.

In this issue of the *JCI*, Ancel, Michaud, and colleagues addressed this unmet need through the combination of nicotinamide and pyridoxine, forms of vitamins B3 and B6, respectively (3). The authors used a high-content imaging screen to identify the two molecules as promoters of proliferation and differentiation in primary human myogenic progenitors from older donors. Intriguingly, forms of both vitamins are being studied to help physical fitness and day-to-day movement in conditions of muscle loss (4, 5). Ancel, Michaud, and authors then used multiple models of muscle damage and aging to articulate the effects of the two compounds on MuSCs. The compounds appeared highly complementary, as nicotinamide stimulated proliferation and pyridoxine promoted differentiation. Indeed, in mice the two compounds synergized in vivo to mitigate MuSC dysfunction and regenerative failure in the aging muscle. Remarkably, in vitro the compound combination rescued the markers of aging-related dysfunction displayed by primary myogenic progenitors isolated from older individuals of both sexes. Finally, in a cohort of 186 randomly selected older men aged 60 years and above, Ancel, Michaud, and authors found correlations between the declines in circulating nicotinamide and pyridoxine levels and the declines in appendicular lean mass and gait speed, two clinical parameters associated in turn with aging-related sarcopenia and mortality (3, 6, 7). It is important to consider that these compounds have very likely parallel or converging effects in myofibers, in addition to MuSCs. Nicotinamide resupplementation through nicotinamide riboside elicited antisarcopenic effects and transcriptomic changes in the aging muscle (8), and the rate-limiting enzyme for nicotinamide, Nampt, is genetically required by myofibers to accrue mass and handle calcium (9). On the other hand, pyridoxine deficiency might be directly involved in muscle contractility issues like intermittent muscle spasms, as recently highlighted in a clinical case of a patient with diabetes (10). Coming back to the paper on hand by Ancel, Michaud, and colleagues, it still quite plausible that skeletal myofibers contribute quite sizably to the correlations of nicotinamide and pyridoxine with lean mass and gait speed (3). While this point was not resolved directly by this report, future studies are warranted to identify different versus convergent effects of those nutraceuticals on MuSCs and myofibers, as this information will likely inform putative clinical applications (3).

## Open questions and conclusions

With their promising results, Ancel and colleagues (3) raise compelling questions for the field moving forward. Notably nicotinamide mainly acted not through its NAD^+^ provision role, but rather through inhibiting CK1α and thereby promoting β-catenin transcriptional outputs. However, the absence of NAD^+^-related benefits is puzzling, as this molecule or its precursor nicotinamide riboside have previously shown benefits on MuSC function in muscular dystrophy and aging (11). Moreover, genetic evidence pinpointing the requirement and sufficiency of CK1α and the WNT/β-catenin pathway for the nicotinamide effects is still missing. This link is particularly critical, considering that inhibitors or degraders of CK1α are typically sought after as antiproliferative agents in cancer (12). Regenerative stimulation and cancer prevention and treatment are often at odds with each other, as they typically are geared toward generating opposite effects on cell proliferation. This caveat becomes quite critical in the context of aging, where on one hand regenerative potential needs to be rescued in critical tissues like muscle, but where on the other hand the risk of cancer-related morbidity is per se dramatically higher (13). Additional studies to shed light on this potentially critical risk balance will support clinical translation for our ever-aging population.

Ancel, Michaud, and authors found that pyridoxine stimulated transcription and phosphorylation of AKT in MuSCs (3). However, the specific components and posttranslational modifications of the complex AKT pathway that are required by pyridoxine to promote MuSC differentiation remain unknown. Determining the required AKT-related mechanisms for the antisarcopenic action of pyridoxine is important because dysfunctions in AKT signaling are common markers of aging (14).

More broadly, regarding the mechanisms of action of nicotinamide and pyridoxine, the question of how much the effects on in vivo muscle regeneration depend on the MuSCs versus the existing myofibers versus other nonmyocyte compartments remains open. MuSC- versus myocyte-specific genetic manipulations will be critical to dissect this point in future projects with those compounds. In this regard, it is also tantalizing to ask whether the two compounds affect inflammation in the contexts of sarcopenic muscle homeostasis and regeneration.

In the human cohort, the aging-related declines in circulating levels of nicotinamide and pyridoxine open two fascinating questions: why is aging reducing those molecules, and is this aging effect dependent only on muscle? Those questions are warranted by the indirect evidence in Ancel et al. (3), indicating that absorption and storage of both compounds appeared stable in older individuals. Those questions will also be crucial to deepening the importance of both molecules not only as antiaging intervention, but also as biomarkers of sarcopenia.

**Figure 1:**
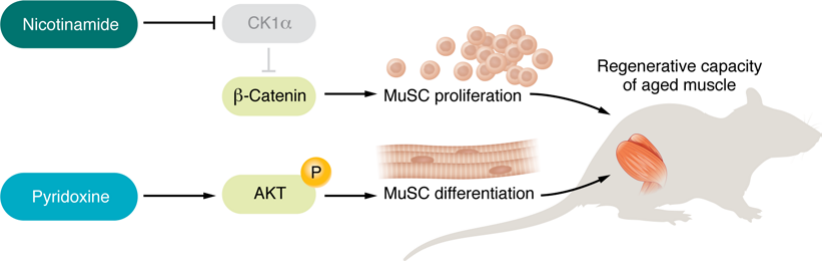
Nicotinamide and pyridoxine converge to rejuvenate aging MuSCs. Ancel et al. (3) used mouse models, human myogenic progenitor cells, and data from the Bushehr Elderly Health cohort to demonstrate the involvement of nicotinamide and pyridoxine with MuSCs. The two nutraceuticals enhanced the regenerative capacity in aging muscle through parallel pathways: nicotinamide stimulated MuSC proliferation via CK1α inhibition, thereby promoting β-catenin transcriptional outputs, and pyridoxine promoted AKT phosphorylation to enhance MuSC differentiation. In aged mice, the treatment combination rescued muscle repair.

In conclusion, despite several key questions left open, the study by Ancel, Michaud, and colleagues unveils a combination of nutraceuticals, suggesting the muscle regenerative capacity can be rejuvenated in the context of aging-related sarcopenia (Figure 1).

## Version 1 12/16/2024

Electronic publication
